# Adapting an emotional regulation and social communication skills group programme to teletherapy, in response to the COVID-19 pandemic

**DOI:** 10.1017/ipm.2020.109

**Published:** 2020-09-11

**Authors:** D. MacEvilly, G. Brosnan

**Affiliations:** St John of God Community Services Ltd, Dublin, Ireland

**Keywords:** CAMHS, CBT, emotional regulation disorders, social communication, telehealth

## Abstract

The COVID-19 pandemic created an unprecedented situation, whereby essential services within child and adolescent mental health services (CAMHS) were suspended. This created a need to modify regular methods of treatment at a rapid pace, to avoid cessation of clinical intervention and prevent potential regression in mental health. Eighteen children with moderate-to-severe mental health disorders and their parents were attending weekly group cognitive behaviour therapy-based sessions (‘The Secret Agent Society’ programme) when the Irish Department of Health suspended face-to-face intervention. This report describes how the group sessions were adapted to individualised, online therapeutic triads between each child, his/her parent and their clinician. Whilst internet technology has emerged as a promising solution to shortfalls in therapy services, in-depth exploration is needed to confirm the efficacy of telehealth for children attending CAMHS.

## Introduction

The Secret Agent Society (SAS) is an espionage themed, multimedia intervention programme, which uses an evidence-based curriculum to improve children’s emotional regulation and social communication skills (Beaumont & Sofronoff, 2008). Developed in Australia, SAS has a substantial international research base supporting its effectiveness for children aged eight to twelve years with a range of neurodevelopmental and anxiety disorders (Thomson *et al*. 2015; Sauve *et al*. 2018; Beaumont *et al*. 2019). Based on the principles of cognitive behaviour therapy (CBT), SAS teaches children how to recognise emotions in themselves and others, express feelings in appropriate ways, talk and play with others, solve social problems and manage bullying. The programme combines multiple therapeutic modalities including weekly group sessions for children, group parent training sessions, a teacher training session, online access to the SAS computer game, home/school practice missions and daily skill tracker cards, all of which help to generalise children’s learning outcomes into real-life experience. The intervention consists of nine distinct topics (Table 1). SAS has been used to support children with complex difficulties associated with Attention Deficit Hyperactivity Disorder, Autism Spectrum Disorder (ASD) and Anxiety Disorders. Statistically significant improvements were reported in the emotional regulation and social communication skills of fifty-one children attending SAS groups in an Irish child and adolescent mental health service (CAMHS) (MacEvilly *et al*. 2019).

**Table 1.** Content of SAS Small Group Programme

Session	Topic
1	Detecting emotions in others
2	Detecting emotions in ourselves
3	Relaxation gadgets
4	Solving friendship problems
5	Conversation skills
6	Playing with others
7	Coping with mistakes
8	Detecting the difference between accidents, jokes and nasty deeds
9	Preventing and managing bullying

SAS is traditionally delivered over nine weekly sessions in small groups of approximately six children. It was being delivered in this way within a CAMHS setting prior to the onset of the COVID-19 pandemic, which subsequently led to the immediate cessation of face-to-face therapeutic contact. Children continued to show significant and at times escalating emotional regulation difficulties at home. Therefore, an alternative method of service delivery was needed for the duration of the COVID-19 crisis. A review of the literature revealed several variations in SAS small group delivery, but none were deemed appropriate given the therapeutic restrictions created by the pandemic. For example, Weiss *et al*. (2018) examined the efficacy of individually delivered SAS intervention to improve mental health difficulties in sixty-eight Canadian children with ASD, aged eight to twelve. Significant improvements in the children’s emotional regulation were noted. This one-to-one format was not an option for the authors in the current global crisis as it entailed face-to-face delivery. In another study, Sofronoff *et al*. (2015) trained parents of forty-one Australian children with ASD to be the principal facilitators of their child’s SAS programme, using an instructional DVD and weekly phone/Skype support. However, during this mode of delivery, there was no interaction between clinician and child, as it was entirely parent led. Published SAS studies did not reflect the challenge faced by the current authors who sought to provide SAS directly to both the child *and* his/her parent, via real-time, interactive, individualised teletherapy.

There is a growing body of both national and international evidence supporting the effectiveness and feasibility of teletherapy for specialist interventions (Gloff *et al*. 2015; Knutsen *et al*. 2016). The benefits of TeleMental Health were also recently highlighted in Ireland’s Mental Health Reform eMental Health document (Cullen, 2018). The report refers to improved access for those reluctant to avail of more traditional forms of therapeutic service, higher cost efficiency and increased user involvement and empowerment. CBT in particular has been successfully delivered to children via telehealth for a range of diverse diagnoses such as depression (Nelson *et al*. 2003), anxiety disorders (Rooksby *et al*. 2015), obsessive–compulsive disorders (Storch *et al*. 2011) and tic disorders (Himle *et al*. 2012). The evidence base supporting the delivery of speech and language intervention to primary school children via telehealth is also growing rapidly (Fairweather *et al*. 2016; Wales *et al*. 2017). However, research into the provision of social communication skills intervention via telehealth is limited and centres predominantly around the feasibility of parent training. Examples of this include Hanen parent training programmes (Comeau, 2020), parent-mediated intervention for children with ASD (Ingersoll *et al*. 2017; Hao *et al*. 2020) and parent–child interaction therapy (Comer *et al*. 2017). A study by Rietdijk *et al*. (2019) ‘indicated potential’ for using telehealth to provide social communication skills training to adults with traumatic brain injury and their families. However, there is a paucity of evidence for the efficacy of social communication intervention delivered remotely to children attending CAMHS and their parents.

Amid significant changes to clinicians’ work practices and uncertainty about redeployment, the authors aimed to prioritise families’ needs during the sudden treatment gap imposed by COVID-19, while at the same time maintaining SAS programme integrity. It was therefore decided to pilot a new variant of SAS delivery, namely adapting group clinic sessions to an individualised, online therapeutic triad between each child, his/her parent and clinician. Remote delivery of the programme would allow intervention to take place in natural, ecologically valid settings convenient for the children and their families at a time of significant disruption and change. The intervention remains ongoing at the time of writing this report.

### Design

This is a description of an adapted intervention programme designed in response to sudden restrictions imposed on CAMHS clinics due to the COVID-19 pandemic. Clinicians considered the confidentiality requirements of each family, possible behaviour management issues arising from having a number of children online simultaneously and the potential for distractibility by the technology itself. It was therefore decided to offer individual parent and child sessions that were specifically tailored to each child’s family circumstances, in the home setting, where the skills would be used. It was predicted that a therapeutic triad would more effectively support family-centred interactions than an online group format, as the clinician would work in partnership with each child and his/her parent. Given the fact that many families were already familiar with online learning from school, it was anticipated that they would be comfortable with videoconferencing technology.

### Participants

Participants were the existing cohort of families who had already attended three sessions of group SAS in CAMHS prior to clinic closure due to COVID-19. Seventeen out of 18 families agreed to take part in the adapted SAS programme at times/dates convenient to them. One family opted to defer treatment until face-to-face therapy provision became available, due to concerns about their low level of computer skills.

Four clinicians were involved in the adaptation and delivery process. All four clinicians are certified SAS facilitators, three of whom are experienced Senior Speech and Language Therapists. They have delivered face-to-face SAS groups to over one hundred children attending CAMHS to date. The fourth clinician is a Clinical Nurse with a post graduate specialism in CBT.

### Satisfaction measures

Evaluation is an established part of routine SAS intervention. Each child’s parent and teacher had already completed Spence’s *Social Skills Questionnaire – Parent (SSQ-P)* and *Teacher (SSQ-T)* versions (1995) and the *Emotion Regulation and Social Skills Questionnaire – Parent (ERSSQ-P)* and *Teacher (ERSSQ-T)* versions (Beaumont & Sofronoff, 2008) prior to the commencement of face-to-face group sessions. The children had already completed two measures designed to assess their knowledge of anxiety and anger management strategies before the SAS groups commenced, as per routine SAS evaluation protocol. These were *‘James and the Maths Test’* (Attwood, 2004a) and ‘*Dylan is being Teased’* (Attwood, 2004b). Parents and children will be asked to complete their measures again immediately after teletherapy delivery and at three- and six-month follow-up. Teacher questionnaires will not be requested in light of school closures due to the COVID-19 pandemic.

Additional child and parent ratings of session satisfaction were developed by the authors to gauge the effectiveness of the adapted teletherapy. Children and parents will be provided with these following the completion of the remote delivery programme. Children’s measures were designed to be brief and provide an opportunity to give feedback on what the children liked/disliked about the new form of intervention, how they might improve it and what skills they found most valuable. Measures include simple Likert scales (e.g. ‘*I liked the sessions a little….a lot….not very much*’) and visuals (e.g. pictures of happy/sad faces) to support ease of completion. Parent satisfaction measures include a rating of their child’s enjoyment of the teletherapy sessions, information regarding programme effectiveness in terms of everyday use of skills learnt via teletherapy and confidence in supporting their child’s emotional regulation. Space has been provided for parents to identify programme strengths and areas for improvement. As well as the satisfaction measures, clinicians will also carry out a qualitative interview with each parent to obtain in-depth information regarding their experiences of taking part in the adapted intervention.

### Teletherapy programme

#### Part 1: Setting the scene for remote delivery

With the existing research evidence in mind, clinicians telephoned each parent to gauge interest in online delivery of SAS and to ensure that conditions were sufficiently favourable to ensure programme sustainability. Possible barriers to taking part included competing demands on parents’ time due to employment, home-schooling commitments and provision of care for siblings. Additional factors discussed were access to Wi-Fi with a stable internet connection and a sufficiently powerful device, household space and freedom from distraction, as well as possible additional psychosocial stress and health concerns. Parents were informed of the practice of telehealth, its benefits and any risks that might be involved due to technological malfunction and problems with confidentiality.

Parents were given the choice of deferring their child’s involvement in the current SAS group until COVID-19 restrictions were lifted or trialing the adapted treatment format instead. Parents were reassured that they and/or their child could drop out of the adapted delivery at any time and re-engage in face-to-face group intervention when feasible. Parents were reminded that their help with SAS home practice activities remained the best predictor of programme success (Beaumont & Sofronoff, 2008) and that merely taking part in the teletherapy alone without home practice would be unlikely to significantly improve their child’s emotional well-being or social communication skills.

#### Part 2: Programme adaptation

Parents and children’s teachers had already attended face-to-face group information sessions and had been given training on the contents of the programme prior to clinic closure. The children had been given their SAS manuals and visual materials to support SAS skills during their first three group sessions. The need for a coordinated, collaborative approach between children, parents, teachers and clinicians had already been stressed from the outset. However, for remote delivery of SAS to be optimally effective, a number of programme adaptations were required to minimise potential barriers to success.

First, the authors reviewed The American Academy of Child and Adolescent Psychiatry guidelines on delivering telepsychiatry with young people (Myers & Cain, 2008) and the University of Oxford’s document on video consultations (Greenhalgh *et al*. 2020). A written guide about teletherapy was then devised, containing an outline of what parents and children could expect during the online sessions (e.g. that the child and his/her parent would share a screen, that the parent would take the lead to control the technology and minimise distraction). A social story (Gray, 2015) was specifically written to explain the sudden change of SAS delivery to each child and to prepare him/her for teletherapy. SAS therapeutic resources, such as relaxation cards, stress ball and an invisible ink pen, usually given by clinicians during the groups were posted home to each parent to give to their child during the sessions. A practice call was made to each parent in advance of the first online session to check technology. Contingency plans were established regarding what to do if the video link failed (the clinician would contact by phone instead). Informed consent was obtained to ensure parents were aware of the potential hazards of online platforms and attention was drawn to advice from the Data Protection Commission, in line with the Data Protection Act (2018).

Teletherapy session checklists were devised by the authors and merged with existing SAS materials, in combination with weekly consultation meetings between facilitators. This ensured consistency of core therapeutic targets across clinics/between clinicians and allowed for adapted treatment fidelity. Sessions were designed to last approximately 45 minutes to maximise children’s concentration and motivation levels. The first session was designed to re-establish rapport with the child in a relaxed, fun online atmosphere while familiarising him/her with the materials provided prior to clinic closure. For subsequent sessions, parents were prompted to make a visual schedule for their child to tick off as each task was completed. A brief ‘tech check’ occurred before commencing each session. Parents were expected to participate in all of the CBT based and social communication tasks which took place during the sessions. A token behaviour modification system had already been established with parents prior to starting the face-to-face groups and this system continued throughout remote delivery. Each child worked towards a small, home-based reward, agreed with parent and clinician at the outset of each session. Sessions progressed from revising preliminary SAS goals with each parent and child (such as recognising and labelling emotions in self and others) to more complex goals such as adjusting responses to difficult emotions using emotional regulation strategies. Goals for the next session were chosen in collaboration with each parent and child and guided by the SAS manual. Types of individualised treatment goals included rating the intensity of emotions on colourful visual paper scales supplied, practising the use of coping strategies for unpleasant feelings, identifying negative thoughts and replacing them with more helpful alternatives. Each child completed tasks during and between sessions in his/her SAS workbook with parental support. Parents were encouraged to continue to prepare for each session by reading their own parent manual in advance and were reminded of the importance of incidental teaching to optimise skill transfer from online sessions to everyday life. Consideration was given to issues pertaining to the social confinement imposed due to COVID-19, such as an increase in children’s aggressive meltdowns, a decrease in predictable routines and new demands of home schooling. A summary of adaptations made to the face-to-face group programme is shown in Table 2.

**Table 2.** Summary of adaptations made to the face-to-face group programme

Adaptations	Group programme	Teletherapy
Format	Face to face	Remote delivery
Participants	Six children per group	1 child + 1–2 parents
Facilitators	Two per group	1–2 facilitators
Children’s Sessions	9	9
Frequency	Weekly	Weekly – accommodating family schedules
Duration	90 minutes	45 minutes
Parent Sessions	Four parent training groups	Four phonecalls to parents
Family	Siblings not involved	Siblings involved if parents wished
School	Teacher involved	Teacher not involved

#### Part 3: Expected outcomes

The COVID-19 pandemic created an unprecedented situation in terms of interruption of essential services within CAMHS. This resulted in the need to modify traditional therapeutic methods at a rapid pace, thereby avoiding cessation of intervention and potential regression in children’s mental health. The innovative use of internet technology has emerged as a promising solution to shortfalls in therapy services, such as waiting times and travel restrictions (Dew *et al.* 2013). However, little is known about the effectiveness of supporting *both* emotional regulation and social communication skills through telehealth.

This report extends related literature by adapting the clinic-based SAS group programme to an individualised, online therapeutic triad between each child, his/her parent and clinician. As the intervention is still ongoing, it is not possible to report on outcomes. However, expected outcomes are similar to face-to-face therapy sessions, such as reduction in children’s emotional dysregulation, increased parental confidence in supporting children’s social communication and greater child reported knowledge of strategies to manage anger/anxiety. It is anticipated that there will be increased parent, child and clinician confidence in working together through an online platform, leading to enhanced co-production between clinic and home. Providing therapy through videoconferencing could become a normalised method of treatment delivery. Consideration may also be given to offering this mode of teletherapy to children who are reluctant/unable to attend CAMHS and to families living in remote areas.

Anticipated challenges with online delivery are that it may be difficult for clinicians to pick up on subtle changes in children’s emotions through a screen and there is no face-to-face peer role modelling. The parent will be relied upon to act as a co-therapist at times, such as during role-plays and brainstorming. The social, supportive elements of both the children’s and parent group sessions will not be possible to replicate (e.g. team games, informal chats at break time). Children’s teachers were not involved in the telehealth delivery as schools were closed. Technology may create its own barriers, such as inadequate bandwidth or unreliable connectivity. However, despite these limitations, it is anticipated that remote delivery of SAS will emerge as an effective, empowering way of providing alternative methods of essential care to children and families during a global emergency and beyond.

## Conclusion

With careful planning and consultation with families, adapting an established face-to-face emotional regulation and social communication programme to teletherapy can be achieved quickly in an unprecedented time. Overall, telehealth is a cost-effective method of supporting continuity of care for families living in geographically remote areas or those reluctant/unable to utilise traditional mental health services. In-depth exploration and analysis of outcome measures are needed to confirm the efficacy of delivering the SAS programme remotely to children with moderate-to-severe mental health disorders. If this pilot intervention suggests that teletherapy has a legitimate place in CAMHS, it may provide a foundation for a randomised control trial comparing face-to-face SAS intervention with remote delivery.
